# Photoexcited State Dynamics and Singlet Fission in Carotenoids

**DOI:** 10.1021/acs.jpca.2c07781

**Published:** 2023-01-26

**Authors:** Dilhan Manawadu, Timothy N. Georges, William Barford

**Affiliations:** † Department of Chemistry, Physical and Theoretical Chemistry Laboratory, University of Oxford, Oxford OX1 3QZ, United Kingdom; ‡ Linacre College, University of Oxford, Oxford OX1 3JA, United Kingdom; ¶ Brasenose College, University of Oxford, Oxford OX1 4AJ, United Kingdom

## Abstract

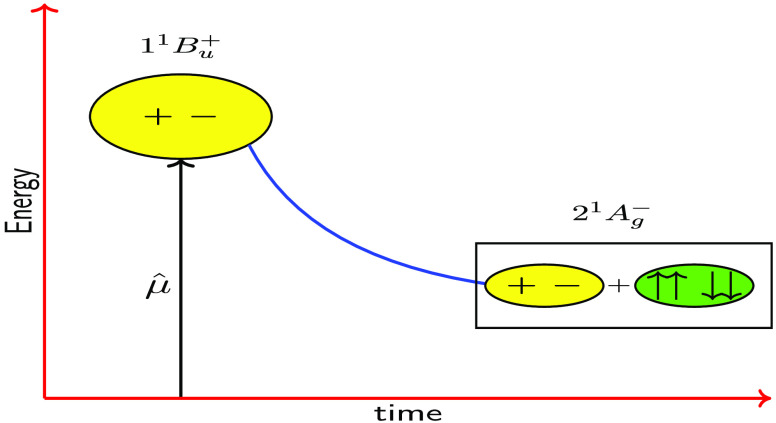

We describe our simulations of the excited state dynamics of the carotenoid neurosporene, following its photoexcitation into the “bright” (nominally 1^1^B_u_^+^) state. To account for the experimental and theoretical uncertainty in the relative energetic ordering of the nominal 1^1^B_u_^+^ and 2^1^A_g_^–^ states at the Franck–Condon point, we consider two parameter sets. In both cases, there is ultrafast internal conversion from the “bright” state to a “dark” singlet triplet-pair state, i.e., to one member of the “2A_g_” family of states. For one parameter set, internal conversion from the 1^1^B_u_^+^ to 2^1^A_g_^–^ states occurs via the dark, intermediate 1^1^B_u_^–^ state. In this case, there is a cross over of the 1^1^B_u_^+^ and 1^1^B_u_^–^ diabatic energies within 5 fs and an associated avoided crossing of the S_2_ and S_3_ adiabatic energies. After the adiabatic evolution of the S_2_ state from predominately 1^1^B_u_^+^ character to predominately 1^1^B_u_^–^ character, there is a slower nonadiabatic transition from S_2_ to S_1_, accompanied by an increase in the population of the 2^1^A_g_^–^ state. For the other parameter set, the 2^1^A_g_^–^ energy lies higher than the 1^1^B_u_^+^ energy at the Franck–Condon point. In this case, there is cross over of the 2^1^A_g_^–^ and 1^1^B_u_^+^ energies and an avoided crossing of the S_1_ and S_2_ energies, as the S_1_ state evolves adiabatically from being of 1^1^B_u_^+^ character to 2^1^A_g_^–^ character. We make a direct connection from our predictions to experimental observables by calculating the time-resolved excited state absorption. For the case of direct 1^1^B_u_^+^ to 2^1^A_g_^–^ internal conversion, we show that the dominant transition at ca. 2 eV, being close to but lower in energy than the T_1_ to T_1_^*^ transition, can be attributed to the 2^1^A_g_^–^ component of S_1_. Moreover, we show that it is the charge-transfer exciton component of the 2^1^A_g_^–^ state that is responsible for this transition (to a higher-lying exciton state), and not its triplet-pair component. These simulations are performed using the adaptive tDMRG method on the extended Hubbard model of π-conjugated electrons. The Ehrenfest equations of motion are used to simulate the coupled nuclei dynamics. We next discuss the microscopic mechanism of “bright” to “dark” state internal conversion and emphasize that this occurs via the exciton components of both states. Finally, we describe a mechanism relying on torsional relaxation whereby the strongly bound intrachain triplet-pairs of the “dark” state may undergo interchain exothermic dissociation.

## 1 Introduction

Carotenoids are a class of linear polyenes of high natural abundance. Carotenoids found in photosynthetic systems serve the dual functions of enhancing their light harvesting properties by absorbing photons in the visible spectrum not accessible to chlorophylls and protecting the light harvesting complexes from excess light.^1,2^ The study of carotenoid photophysics is important for understanding their functions in photosynthetic systems.^3,4^

The quasi-one-dimensional nature of polyenes enhances electron–electron interactions and electron–nuclear coupling, and gives rise to a complex excited state structure.^5−12^ In 1972, it was observed that in polyenes there exists a symmetry-forbidden 2^1^A_g_^–^ “dark” excited state (usually labeled S_1_) below the photoexcited 1^1^B_u_^+^ state (usually labeled S_2_).^5,6^ Then, in 1987, it was shown that there exist other dark states within the S_2_–S_1_ gap.^8^ Upon photoexcitation to the S_2_ state, these dark excited states are involved in ultrafast internal conversion processes, giving rise to the exotic photophysical properties of polyenes, including singlet fission.

Singlet fission is a photophysical process by which a singlet photoexcited state dissociates into two spin uncorrelated triplets. In carotenoids, the first step of singlet fission is understood to be the internal conversion from the photoexcited 1^1^B_u_^+^ state to a correlated singlet triplet-pair state. The mechanism of this internal conversion process has been heavily debated.^13^ Spectroscopic studies of carotenoid excited states reveal that although the S_2_–S_1_ gap increases with conjugation length, the S_2_ lifetime behaves nonmonotonically: initially increasing and then decreasing with conjugation length, in an apparent violation of the energy gap law. This implies for longer carotenoids, as for polyenes, that an intermediate dark state exists which might be involved in the internal conversion process.^14,15^

Recent theoretical work using diabatic models continues to provide evidence for the importance of the low-lying dark excited states of polyenes to their photophysics, especially in the singlet fission process.^16^ However, *ab initio* calculations of polyene excited states argue that nuclear reorganization following photoexcitation can facilitate the internal conversion process, without needing to invoke intermediate dark states.^17,18^

In a theoretical and computational study using the density matrix renormalization group (DMRG) method to solve the Pariser–Parr–Pople–Peierls (PPPP) model of π-conjugated electrons, Valentine et al.^19^ showed that the dark excited states, 2^1^A_g_^–^, 1^1^B_u_^–^, 3^1^A_g_^–^, etc., belong to the same family of fundamental excitation with different center-of-mass kinetic energies. The triplet-pair nature of this 2A_g_ family (or band) of states was established by calculating the spin–spin correlation, bond dimerization, and triplet-pair overlaps.

In a recent paper we described our dynamical simulations of singlet triplet-pair production in photoexcited zeaxanthin using the adaptive time-dependent DMRG (tDMRG) method and Ehrenfest dynamics.^20^ We chose a parameter regime where at the Franck–Condon point the diabatic energies satisfy *E*(2^1^A_g_^–^) < *E*(1^1^B_u_^+^) < *E*(^1^B_u_^–^), while the adiabatic singlet states are S_1_ ≈ 2^1^A_g_^–^, S_2_ ≈ 1^1^B_u_^+^, and S_3_ ≈ 1^1^B_u_^–^. Within 5 fs of photoexcitation to S_2_, there is a diabatic crossover of the 1^1^B_u_^+^ and 1^1^B_u_^–^ energies but an avoided crossing of the S_2_ and S_3_ energies, such that S_2_ evolves quasi-adiabatically from the 1^1^B_u_^+^ state to the 1^1^B_u_^–^ state. Since zeaxanthin possesses *C*_2_ symmetry, there is no further interstate conversion from the 1^1^B_u_^–^ to the 2^1^A_g_^–^.

In this paper, we extend that work to investigate internal conversion in neurosporene, a molecule of 18 conjugated carbon atoms that does not possess *C*_2_ symmetry, thus permitting 1^1^B_u_^+^ to 2^1^A_g_^–^ state conversion. We consider two parameter sets, (a) *E*(2^1^A_g_^–^) < *E*(1^1^B_u_^+^) < *E*(^1^B_u_^–^) at the Franck–Condon point, where internal conversion from the 1^1^B_u_^+^ to 2^1^A_g_^–^ states occurs via the intermediate 1^1^B_u_^–^ state and (b) *E*(1^1^B_u_^+^) < *E*(2^1^A_g_^–^) < *E*(^1^B_u_^–^) at the Franck–Condon point, where there is direct internal conversion from the 1^1^B_u_^+^ to 2^1^A_g_^–^ states. In both cases, we show that after 50 fs the yield of the singlet triplet-pair states is ca. 65%.

As well as describing the dynamical simulations of internal conversion, this paper has three further goals. First, we discuss our calculated transient absorption spectra from the evolving state, and we use our results to interpret experimental observations. In particular, we argue that a dominant absorption feature at ca. 2 eV, close to but lower in energy than a triplet state absorption, originates from the charge-transfer exciton component of the “dark” 2^1^A_g_^–^ state. Second, using the theory of the “dark” state of ref (21), where it was shown that this state contains both singlet triplet-pair and charge-transfer exciton character, we describe the microscopic mechanism of “bright” to “dark” state internal conversion in carotenoids. Finally, we examine the question of whether the bound intrachain triplet-pairs can undergo exothermic interchain dissociation. We show that this is possible if interchain transfer is accompanied by torsional relaxation, implying that the carotenoid dimers should have a twisted ground state geometry.

A companion paper^22^ describes our computational DMRG methodology for simulating the excited state dynamics of strongly correlated electron systems. It also analyses in greater detail the physics of the diabatic crossover and the adiabatic avoided crossing.

The contents of this paper are the following. After briefly introducing our model in section 2, section 3 describes our dynamical simulations of photoexcited state interconversion, as well as our calculation and interpretation of the transient excited state absorption. Section 3 concludes with a microscopic explanation of the ultrafast “bright” to “dark” interstate conversion observed in carotenoids. Section 4 shows that interchain singlet dissociation into triplets can be exothermic if accompanied by torsional relaxation. We conclude in section 5.

## 2 Computational Methods

The π-electron system is described by the extended Hubbard (or UV) model, defined by
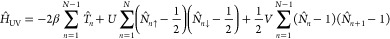
1where *n* labels the *n*th C atom, *N* is the number of conjugated C atoms, and *N*/2 is the number of double bonds.
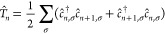
is the bond order operator, *ĉ*_*n*,σ_^†^ (*ĉ*_*n*,σ_) creates (destroys) an electron with spin σ in the p_*z*_ orbital of the *n*th C atom, and *N̂*_*n*_ is the number operator. *U* and *V* correspond to Coulomb parameters, which describe interactions of two electrons in the same orbital and nearest neighbors, respectively, and β = 2.4 eV represents the electron hopping integral between neighboring C atoms.

The electron–nuclear coupling is described by
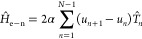
2where α = 4.593 eV Å^–1^ is the electron–nuclear coupling parameter and *u*_*n*_ is the displacement of the *n*th C atom from its undimerized geometry. The nuclear degrees of freedom are described by the classical Hamiltonian
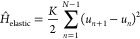
3where *K* = 46 eV Å^–2^ is the nuclear spring constant.

To project out the high spin eigenstates of the Hamiltonian, we complement the Hamiltonian with
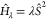
4where *Ŝ* is the total spin operator and λ > 0.

The UV-Peierls (UVP) Hamiltonian, defined by
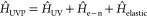
5is invariant under a particle–hole transformation. For idealized carotenoid structures with 2-fold rotation symmetry, its eigenstates will have definite *C*_2_ and particle–hole symmetries.^12^ Therefore, its eigenstates are labeled either A_g_^±^ or B_u_^±^. We define the eigenstates of (*Ĥ*_UVP_ + *Ĥ*_λ_) as diabatic states. The ordering of these states is highly sensitive to the *U* and *V* parameters. It was shown in ref (20) that for the UVP model with *U* = 7.25 eV and *V* = 2.75 eV, the 1^1^B_u_^–^ relaxed energy is lower than the 1^1^B_u_^+^ relaxed energy for chain lengths *N* ≥ 10, while the 1^1^B_u_^–^ vertical energy is higher than the 1^1^B_u_^+^ vertical energy for *N* ≤ 22. Both the vertical and relaxed 2^1^A_g_^–^ energies are below the 1^1^B_u_^+^ energies for all relevant chain lengths. This implies that for these model parameters internal conversion from the 1^1^B_u_^+^ state to the 2^1^A_g_^–^ state is expected to proceed via the 1^1^B_u_^–^ state for 10 ≤ *N* ≤ 22.

Recent theoretical studies using highly accurate ab initio methods, however, suggest that internal conversion in carotenoids from the 1^1^B_u_^+^ state to the 2^1^A_g_^–^ state can proceed directly, without involving intermediate dark states.^17,18^ In order to investigate this particular mechanism, we choose a second set of *U* and *V* parameters, namely *U* = 7.25 eV and *V* = 3.25 eV. The vertical and relaxed excitation energies for this parametrization are illustrated in Figure S1 of the Supporting Information. For this parameter regime, we find that for all relevant chain lengths the 2^1^A_g_^–^ relaxed energy is lower than the 1^1^B_u_^+^ relaxed energy, and that the 2^1^A_g_^–^ vertical energy is higher than the 1^1^B_u_^+^ vertical energy. Therefore, direct internal conversion from the 1^1^B_u_^+^ state to the 2^1^A_g_^–^ state is energetically possible for this parameter set.

Since *Ĥ*_UVP_ is invariant to particle–hole exchange, a symmetry breaking term *Ĥ*_ϵ_ is introduced into our model to facilitate internal conversion from the 1^1^B_u_^+^ state, which has positive particle–hole symmetry, to the triplet-pair states, which have negative particle–hole symmetry. This term is given by
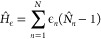
6where ϵ_*n*_ is the potential energy on the *n*th C atom.

We denote the *i*th singlet eigenstate of the full Hamiltonian *Ĥ* = (*Ĥ*_UVP_ + *Ĥ*_λ_ + *Ĥ*_ϵ_) as S_*i*_ (where S_0_ is the ground state). We define these to be adiabatic states. We note that for *V* = 2.75 eV (as in ref (20)), S_2_ is taken to be the initial state, Ψ(*t* = 0), at time *t* = 0, as it has the largest 1^1^B_u_^+^ character. Similarly, for *V* = 3.25 eV, Ψ(*t* = 0) = S_1_, as it has the largest 1^1^B_u_^+^ character.

As described in ref (22), *Ĥ*_ϵ_ is optimized under the constraint ϵ_*n*_ < ϵ_max_ such that the ground state π-electron density on the *n*th C atom reproduces the Mulliken charge densities of the π-system found via ab initio density functional theory (DFT) calculations. The optimized *Ĥ*_ϵ_ is given in Table S1 in the Supporting Information. The cutoff ϵ_max_ = 1.0 is chosen such that Ψ(*t* = 0) retains sufficient 1^1^B_u_^+^ character, while accurately reproducing the DFT densities.

The electronic states and the ground state equilibrium geometry are determined via the static DMRG method, while the time evolution of the initially prepared photoexcited singlet is determined via adaptive tDMRG.^23,24^ The nuclear degrees of freedom are treated classically via the Ehrenfest equations of motion. The theoretical and computational techniques employed here are described in full detail in the accompanying methodology paper.^22^

## 3 “Bright” to “Dark” State Internal Conversion

We begin our discussion of “bright” to “dark” state internal conversion by describing our dynamical simulations in section 3.1. These simulations predict that interstate conversion does happen within 10 fs. In section 3.2 we then explain how interstate conversion occurs and why it is so fast.

### 3.1 Computational Results

**Figure 1:**
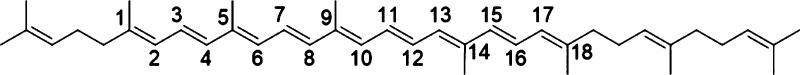
Structural formula of neurosporene, illustrating the 18 C atom (9 double-bond) π-conjugated system.

All of our calculations are performed on the carotenoid neurosporene, whose chemical formula is illustrated in Figure 1. As neurosporene does not possess *C*_2_ symmetry, transitions from B_u_ to A_g_ states are not symmetry forbidden. This gives rise to a complex dynamical relaxation process involving the 1^1^B_u_^+^, 1^1^B_u_^–^, and 2^1^A_g_^–^ diabatic states. This is in contrast to our previous work on zeathanxin,^20^ which does possess *C*_2_ symmetry, and therefore only exhibits 1^1^B_u_^+^ to 1^1^B_u_^–^ state interconversion.

#### 3.1.1 Internal Conversion from the 1^1^B_u_^+^ to 2^1^A_g_^–^ States via the 1^1^B_u_^–^ State

**Figure 2:**
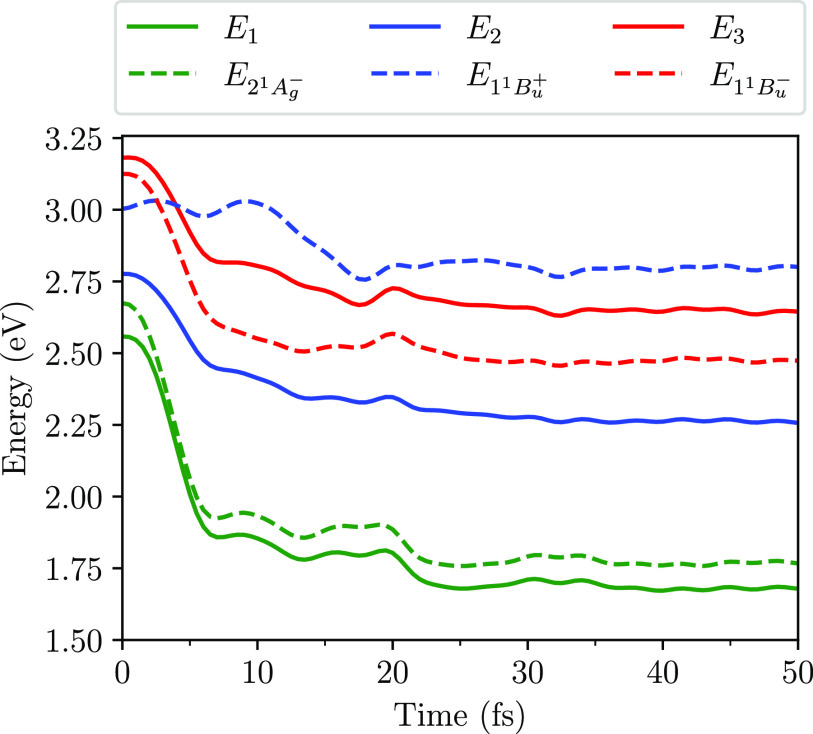
Excitation energies as a function of time of the diabatic 2^1^A_g_^–^, 1^1^B_u_^+^, and 1^1^B_u_^–^ states and the adiabatic S_1_, S_2_, and S_3_ states. These energies are found for neurosporene with *V* = 2.75 eV. The 1^1^B_u_^+^ and 1^1^B_u_^–^ energies exhibit a crossover at ∼5 fs, while the S_2_ and S_3_ energies (i.e., *E*_2_ and *E*_3_, respectively) exhibit an avoided crossing.

For the parameter set *V* = 2.75 eV the initial photoexcited system Ψ(*t*) is prepared in the adiabatic state S_2_, as this has the largest overlap with the diabatic 1^1^B_u_^+^ state at the Franck–Condon point. The nuclei begin in the ground state geometry and experience resultant forces exerted by the electrons, which cause *Ĥ*_e–n_ to change and initiate the evolution of the electronic and nuclear degrees of freedom. Figure 2 shows the calculated adiabatic and diabatic excited energies as a function of time. A crossover of the diabatic 1^1^B_u_^–^ and 1^1^B_u_^+^ energies occurs at ∼5 fs, while the adiabatic S_2_ and S_3_ energies exhibit an avoided crossing, as the coupling between the diabatic states is nonzero, i.e., ⟨1^1^B_u_^+^|*Ĥ*_ϵ_|1^1^B_u_^–^⟩ ≠ 0.

**Figure 3:**
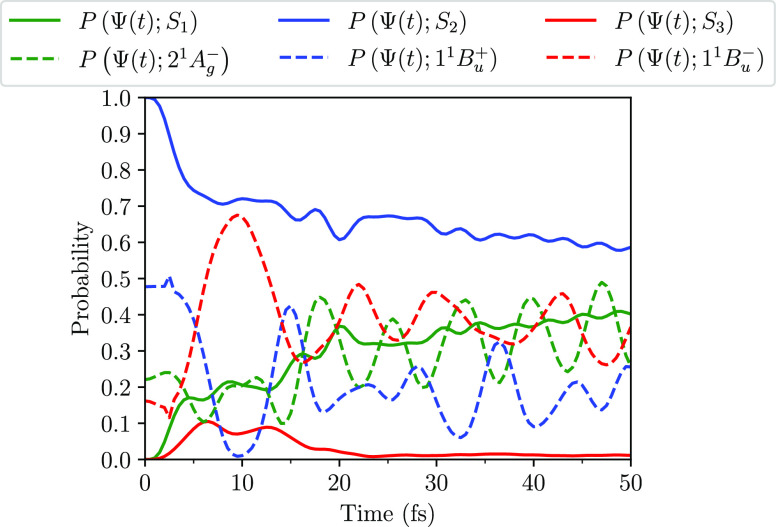
Probabilities as a function of time that the system described by Ψ(*t*) occupies the adiabatic states, S_1_, S_2_, and S_3_, and the diabatic states, 2^1^A_g_^–^, 1^1^B_u_^+^, and 1^1^B_u_^–^. The results are for neurosporene with *V* = 2.75 eV. Note that Ψ(*t* = 0) = S_2_.

The triplet-pair nature of the system at time *t* is determined by calculating the probabilities that the system described by Ψ(*t*) occupies the triplet-pair states, 1^1^B_u_^–^ and 2^1^A_g_^–^. Figure 3 shows the probabilities that Ψ(*t*) occupies the diabatic states 2^1^A_g_^–^, 1^1^B_u_^+^, and 1^1^B_u_^–^, and the adiabatic states, S_1_, S_2_ and S_3_. At the avoided crossing Ψ(*t*) exhibits an adiabatic transition from the 1^1^B_u_^+^ state to the 1^1^B_u_^–^ state. Within 10 fs, Ψ(*t*) predominantly occupies the 1^1^B_u_^–^ diabatic state.

After the ultrafast adiabatic transition to the 1^1^B_u_^–^ state, the system continues to undergo a slow nonadiabatic transition to the 2^1^A_g_^–^ state. This is a consequence of the nonzero coupling between the 1^1^B_u_^–^ and 2^1^A_g_^–^ states, i.e., ⟨1^1^B_u_^–^|*Ĥ*_ϵ_|2^1^A_g_^–^⟩ ≠ 0.

As both 2^1^A_g_^–^ and 1^1^B_u_^–^ are triplet-pair states and noting that *P*(S_3_; Ψ(*t*)) ∼ 0 in the long-time limit, we extend the singlet triplet-pair yield calculation for a two level system^20,22^ to calculate the total triplet-pair state probability, *P*_classical_, as
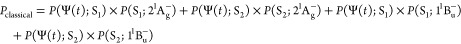
7After ∼50 fs this yield is ∼70%. (The probabilities, *P*(S_*i*_, ϕ_*j*_), that the adiabatic state, S_*i*_, occupies the diabatic state, ϕ_*j*_, are shown in Figure S2 of the Supporting Information.)

**Figure 4:**
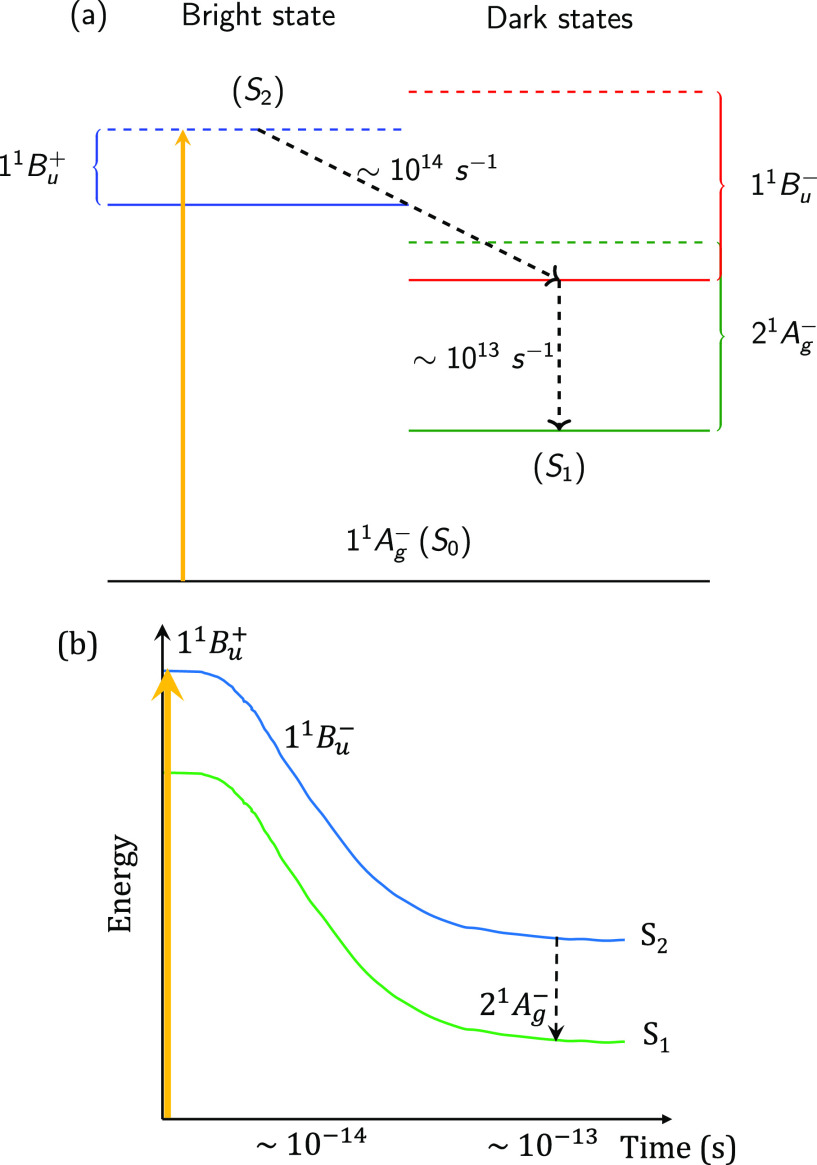
Schematic diagrams illustrating internal conversion between the excited states of neurosporene (using *V* = 2.75 eV). (a) Diabatic state representation: 1^1^B_u_^+^ (blue), 1^1^B_u_^–^ (red), and 2^1^A_g_^–^ (green). The dashed (solid) horizontal lines represent the vertical (relaxed) energies of the states. The approximate rate constants are also indicated. (b) Adiabatic state representation, showing S_2_ evolve adiabatically from 1^1^B_u_^+^ to 1^1^B_u_^–^, before the slower nonadiabatic transition from S_2_ to S_1_. S_2_ (blue) and S_1_ (green).

A scheme illustrating the internal conversion between the excited states is shown in Figure 4 for the case of the intermediate state.

#### 3.1.2 Direct Internal Conversion from the 1^1^B_u_^+^ State to the 2^1^A_g_^–^ State

**Figure 5:**
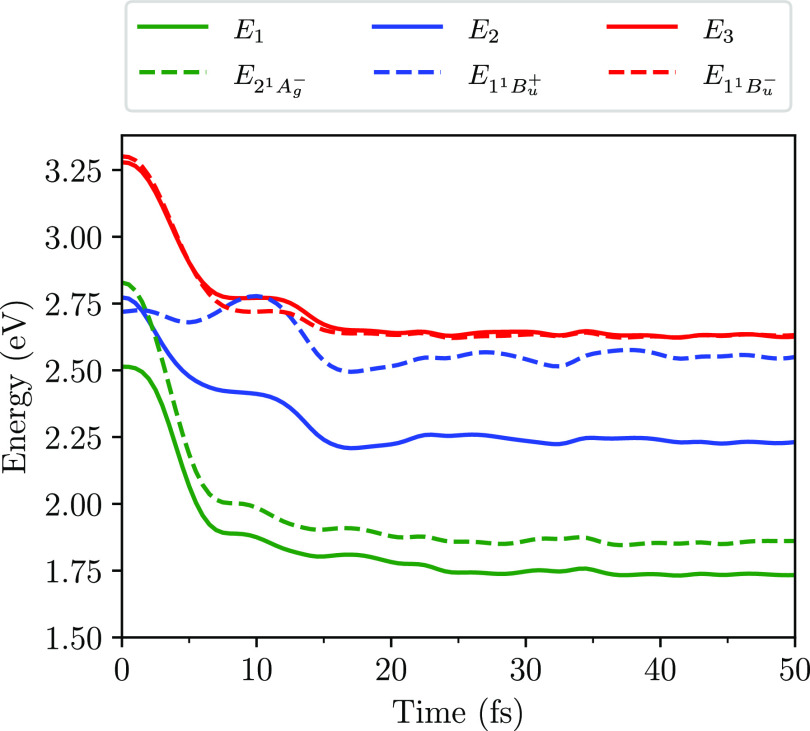
Excitation energies as a function of time of the diabatic 1^1^B_u_^+^, 2^1^A_g_^–^, and 1^1^B_u_^–^ states and the adiabatic S_1_, S_2_, and S_3_ states. These energies are found for neurosporene with *V* = 3.25 eV. The 1^1^B_u_^+^ and 2^1^A_g_^–^ energies exhibit a crossover at ∼3 fs, while the S_1_ and S_2_ energies exhibit an avoided crossing.

As described in section 2, for the parameter set where *V* = 3.25 eV, the primary photoexcited state is S_1_, which is predominately 1^1^B_u_^+^. The singlet adiabatic and diabatic energies as a function of time are illustrated in Figure 5. The 2^1^A_g_^–^ and 1^1^B_u_^+^ energies crossover within ∼3 fs. In contrast, as a consequence of the diabatic coupling of the 2^1^A_g_^–^ and 1^1^B_u_^+^ states, the S_1_ and S_2_ energies display an avoided crossing.

**Figure 6:**
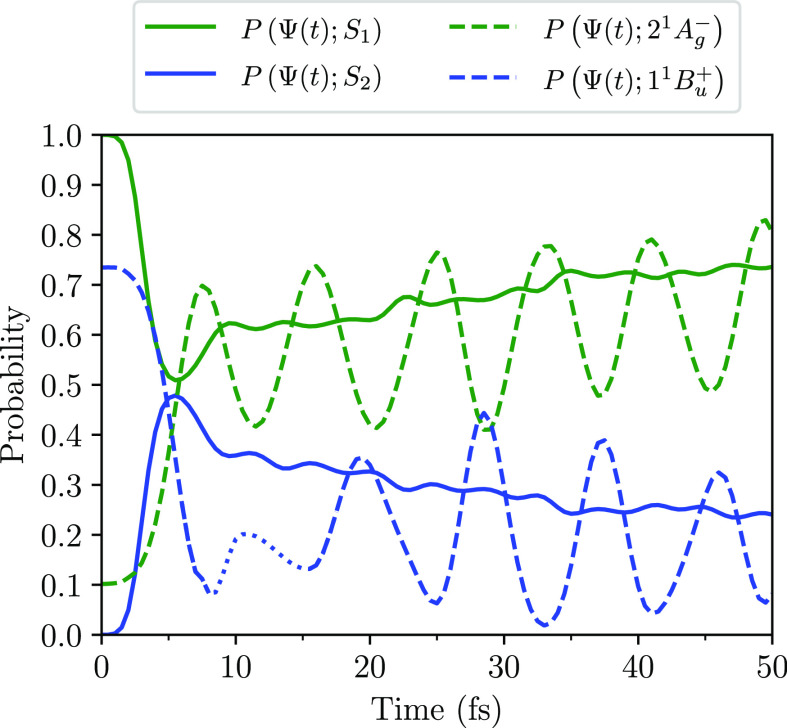
Probabilities as a function of time that the system described by Ψ(*t*) occupies the adiabatic states, S_1_ and S_2_, and the diabatic states, 2^1^A_g_^–^ and 1^1^B_u_^+^. These results are for neurosporene with *V* = 3.25 eV. Note that Ψ(*t* = 0) = S_1_. (The dotted curve for the 1^1^B_u_^+^ occupation is an interpolation of the computed data from 8 to 15 fs, as during this time the 1^1^B_u_^+^ and 1^1^B_u_^–^ energies are quasidegenerate, making it difficult to numerically resolve these wave functions.).

The probabilities that Ψ(*t*) occupies the adiabatic and diabatic states, illustrated in Figure 6, show that the avoided crossing is accompanied by a transition of Ψ(*t*) from the 1^1^B_u_^+^ state to the 2^1^A_g_^–^ state, while predominantly remaining in the S_1_ state. The oscillations of the diabatic populations can be understood by a quasistationary two-state approximation, as discussed in ref (22).

**Figure 7:**
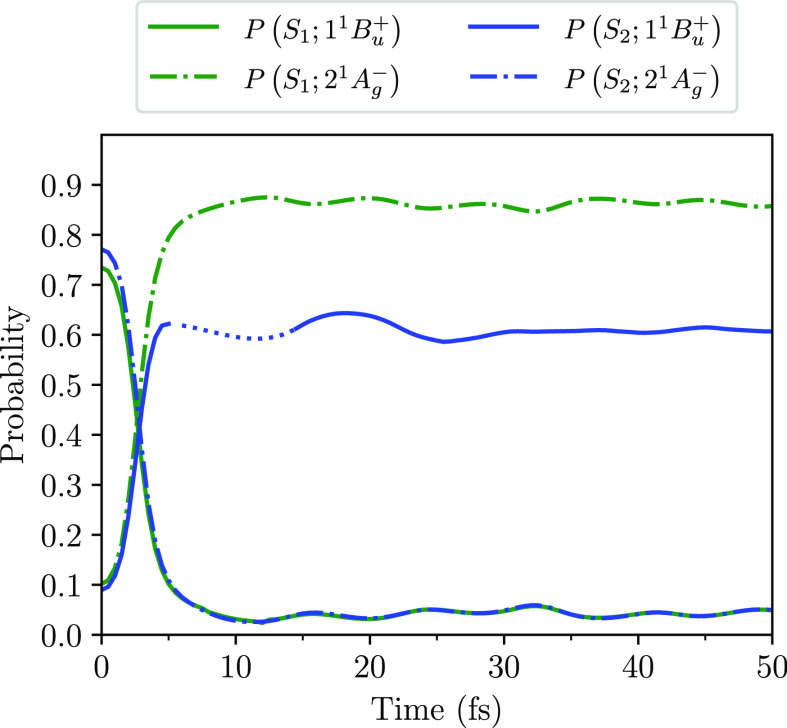
Probabilities as a function of time that the adiabatic states, S_1_ and S_2_, occupy the diabatic states, 2^1^A_g_^–^ and 1^1^B_u_^+^. These results are for neurosporene with *V* = 3.25 eV. (The dotted curve for the 1^1^B_u_^+^ occupation is an interpolation of the computed data from 8 to 15 fs, as during this time the 1^1^B_u_^+^ and 1^1^B_u_^–^ energies are quasidegenerate, making it difficult to numerically resolve these wave functions.).

Further evidence for the adiabatic transition is provided by the calculated probabilities that the adiabatic states occupy the diabatic states, shown in Figure 7. At *t* = 0, the photoexcited state, S_1_, primarily occupies the exciton state, 1^1^B_u_^+^, while S_2_ primarily occupies the triplet-pair state, 2^1^A_g_^–^. After the avoided crossing, at which the diabatic states contribute equally to the adiabatic states, the S_1_ state predominantly occupies the 2^1^A_g_^–^ state, while the S_2_ state predominantly occupies the 1^1^B_u_^+^ state. The 2^1^A_g_^–^ yield after ∼50 fs is ∼60%.

**Figure 8:**
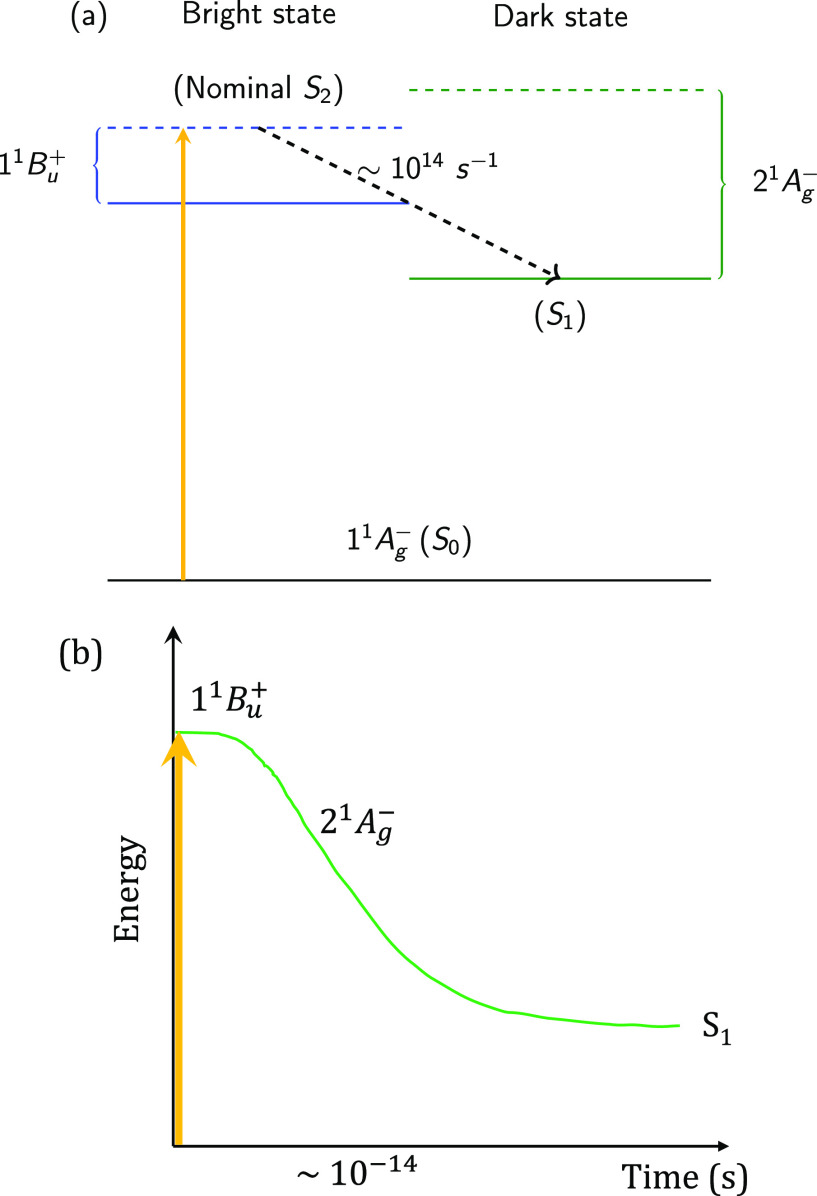
Schematic diagrams illustrating internal conversion between the excited states of neurosporene (using *V* = 3.25 eV). (a) Diabatic state representation: 1^1^B_u_^+^ (blue) and 2^1^A_g_^–^ (green). The dashed (solid) horizontal lines represent the vertical (relaxed) energies of the states. The approximate rate constant is also indicated. (b) Adiabatic state representation, showing S_1_ evolve adiabatically from 1^1^B_u_^+^ to 2^1^A_g_^–^.

A scheme illustrating the internal conversion between the excited states is shown in Figure 8 for the case of no intermediate state.

#### 3.1.3 Transient Excited State Absorption

Transient (i.e., time-resolved) spectroscopy is an important experimental technique used in the study of carotenoid photophysics. Seminal work which utilized transient spectroscopy experiments included the measurement of the S_1_ lifetime of carotenoids,^25^ direct observation of the S_1_ dark state,^26^ and the detection of dark intermediate states between the S_1_ and S_2_ states.^27,28^

The theoretical transient absorption spectrum from state S_*i*_ at time *t* is given by the expression
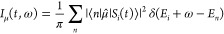
8where *E*_*i*_ is the energy of state S_*i*_. In this section, we present our results for transient excited state absorption calculations using the Lanczos-DMRG method.^29,30^ The computational methodology is described in ref (22).

**Figure 9:**
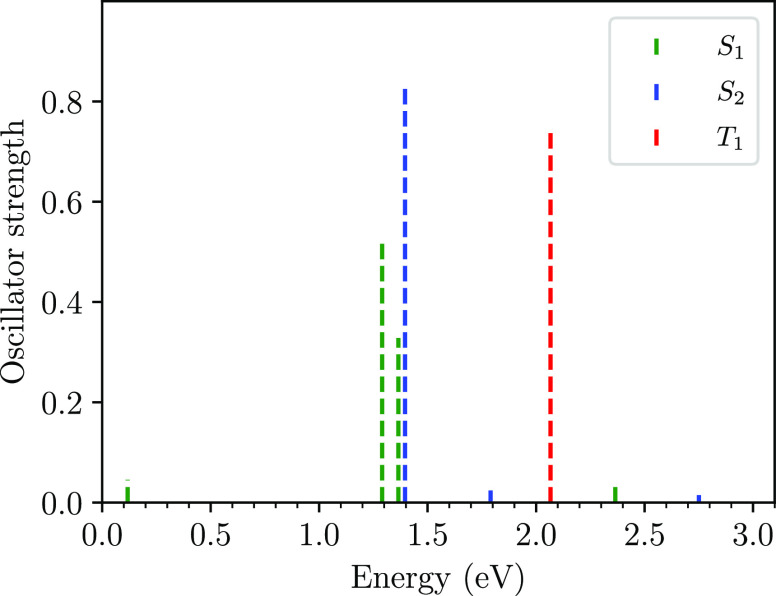
Calculated transient absorption spectra at *t* = 0 from the S_1_ ≡ 1^1^B_u_^+^, S_2_ ≡ 2^1^A_g_^–^, and T_1_ states. Results are for a system with no broken-symmetry, i.e., *Ĥ*_ϵ_ = 0.

We present results for *V* = 3.25 eV, when there is direct 1^1^B_u_^+^ to 2^1^A_g_^–^ state conversion. First, we consider an ideal system with both particle–hole and *C*_2_ symmetries (i.e., we set *Ĥ*_ϵ_ = 0). Thus, at the Franck–Condon point (i.e., at *t* = 0 fs) S_1_ ≡ 1^1^B_u_^+^ and S_2_ ≡ 2^1^A_g_^–^. The calculated initial absorption spectra from S_1_ and S_2_ and the triplet ground state, T_1_, are shown in Figure 9. The three peaks arising from the S_1_ state at 0.1, 1.3, and 1.4 eV are attributed to transitions from the 1^1^B_u_^+^ state to the 2^1^A_g_^–^, 4^1^A_g_^–^, and 5^1^A_g_^–^ states, respectively.

**Figure 10:**
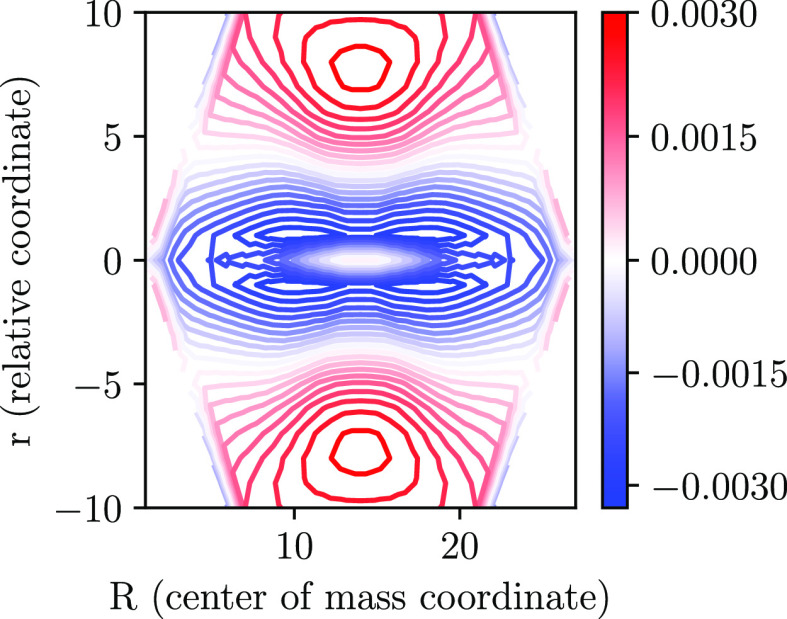
*n*^1^B_u_^+^ exciton wave function calculated using eq 18 of ref (19), using the PPP model with *N* = 54. This has two nodal surfaces along the relative (i.e., electron–hole) coordinate and is thus dipole-connected to the charge-transfer exciton component of the 2^1^A_g_^–^ state, which has one nodal surface along the relative coordinate. See ref (19) for the full parametrization.

The 2^1^A_g_^–^ state is comprised of a singlet triplet-pair component and an odd-parity charge-transfer exciton component (see section 3.2 and refs (19 and 21)). If the absorption signal from the 2^1^A_g_^–^ state arose from a transition from its bound triplet-pair component to T_1_T_1_^*^, we would expect this to be higher in energy than the T_1_ to T_1_^*^ transition.^31^ We note, however, that this absorption peak is significantly lower in energy compared to the lowest absorption from the T_1_ state. In contrast, if the absorption signal from the 2^1^A_g_^–^ state arose from a transition from its charge-transfer exciton component, the resulting *n*^1^B_u_^+^ state would be an even-parity exciton. We verify this latter hypothesis by calculating the exciton wave function of the *n*^1^B_u_^+^ state; this is shown in Figure 10.^a^ By observing the nodal structure of the *n*^1^B_u_^+^ exciton wave function, we conclude that the 2^1^A_g_^–^ to *n*^1^B_u_^+^ transition does indeed arise from the charge-transfer exciton component of the 2^1^A_g_^–^ state to a Mott–Wannier exciton.^12^

Next, we consider the realistic Hamiltonian with the inclusion of the symmetry breaking term, *Ĥ*_ϵ_. For this set of parameters, the 1^1^B_u_^+^ and 2^1^A_g_^–^ energies exhibit a crossover, while the S_1_ and S_2_ energies exhibit an avoided crossing (as shown in Figure 5).

**Figure 11:**
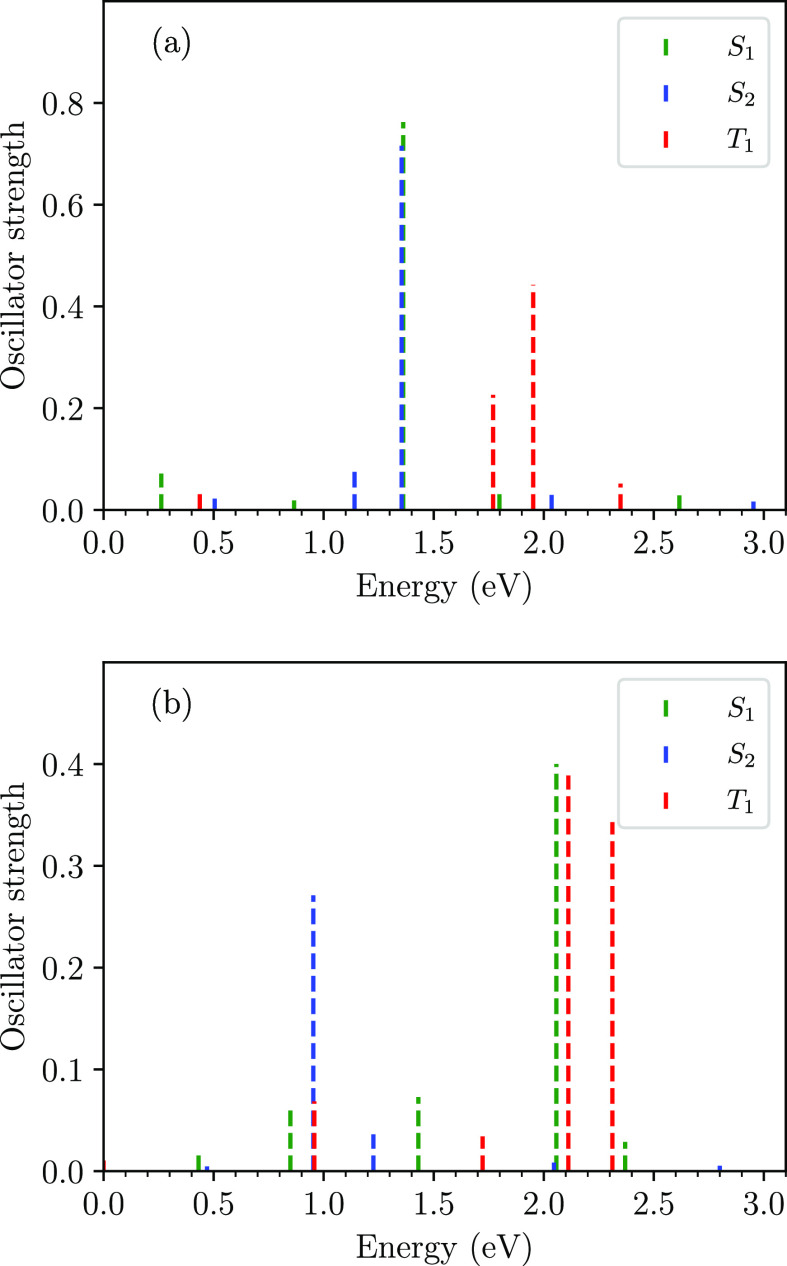
Calculated transient absorption spectra from the S_1_, S_2_, and T_1_ states of neurosporene. (a) At *t* = 0, where S_1_ ≈ 1^1^B_u_^+^ and S_2_ ≈ 2^1^A_g_^–^. Note the overlapping S_1_ and S_2_ absorption at ∼1.4 eV. (b) At *t* = 20 fs, where S_1_ ≈ 2^1^A_g_^–^ and S_2_ ≈ 1^1^B_u_^+^. Here, the singlet state absorption spectra are weighted by *P*(Ψ(*t*); S_*i*_).

The calculated absorption spectra at the Franck–Condon point from the S_1_ and S_2_ states and the triplet ground state, T_1_, are shown in Figure 11a. At *t* = 0 fs, S_1_ predominantly occupies the 1^1^B_u_^+^ state, while S_2_ predominantly occupies the 2^1^A_g_^–^ state. The absorption at ∼0.3 eV corresponds to the S_1_ to S_2_ transition, while the absorption maxima observed around 1.4 eV is the transition from the 1^1^B_u_^+^ state to a high energy ^1^A_g_^–^ state. Due to the inclusion of the symmetry breaking term, the adiabatic state T_1_ will have some diabatic triplet excited state character, and therefore two absorption peaks are observed from T_1_. As for Figure 9, the transition from the charge-transfer exciton component of the 2^1^A_g_^–^ state to the *n*^1^B_u_^+^ state at ∼1.4 eV is lower in energy than the triplet absorption peaks.

As the evolving photoexcited system, Ψ(*t*), reaches equilibrium it occupies both the S_1_ and S_2_ states. Figure 11b shows the weighted absorption spectra at *t* = 20 fs of these states, as well as the absorption spectra of the T_1_ state. After passing through the avoided crossing at ∼3 fs, the S_1_ state now predominantly occupies the 2^1^A_g_^–^ state, while the S_2_ state predominantly occupies the 1^1^B_u_^+^ state. This change of occupations is reflected in the calculated transient spectra, as the major signals originating from the S_1_ state are now higher in energy compared to those originating from the S_2_ state. At the relaxed geometry, the triplet peaks are blue-shifted to 2.1 and 2.3 eV, which are close to the relaxed excited state values previously calculated for polyenes.^19^

As the S_1_ state at 20 fs predominantly occupies the 2^1^A_g_^–^ state, we attribute the absorption peak of the S_1_ state at ∼2.1 eV to a transition from the charge-transfer exciton component of the 2^1^A_g_^–^ state. This energy is blue-shifted from the corresponding transition from the 2^1^A_g_^–^ component of the S_2_ state at *t* = 0, because of the large reorganization energy of the 2^1^A_g_^–^ state. This energy is close to the experimentally observed 2^1^A_g_^–^ to *n*^1^B_u_^+^ transition of carotenoids.^13,25,31^ We also note that the energy of the major absorption from S_2_ around 0.9 eV agrees with the experimentally observed 1^1^B_u_^+^ to *m*^1^A_g_^–^ transition.^32^

In summary, our transient excited state absorption calculations predict the following. At the Franck–Condon point there will be a photoexcited absorption at ca. 1.4 eV resulting from the 1^1^B_u_^+^ to *n*^1^A_g_^–^ transition (and a weak transition at ca. 0.3 eV resulting from the 1^1^B_u_^+^ to 2^1^A_g_^–^ transition). Within 20 fs, however, the adiabatic evolution of S_1_ from 1^1^B_u_^+^ to 2^1^A_g_^–^ character results in a new transition from the charge-transfer exciton component of the 2^1^A_g_^–^ state at ca. 2.0 eV, while a weaker transition at ca. 0.9 eV arises from the residual 1^1^B_u_^+^ component of the evolving wave function.

### 3.2 How and Why Does “Bright” To “Dark” State Internal Conversion Occur?

As we have seen in the previous two sections, as a consequence of diabatic energy-level crossings, internal conversion from the optically “bright” state to the “dark” state occurs within 10 fs. In this section, we address the questions of how and why this process occurs.

As described in ref (21), the 2^1^A_g_^–^ state is a linear combination of a singlet triplet-pair and an odd-parity charge-transfer exciton. Triplet-pair binding occurs because when a pair of triplets occupy neighboring ethylene dimers a one-electron transfer converts them to the odd-parity charge-transfer exciton. This hybridization causes a nearest neighbor triplet–triplet attraction. Similarly, the 1^1^B_u_^+^ state is predominately a Frenkel exciton (i.e., an electron–hole bound on the same dimer). However, the 1^1^B_u_^+^ state also consists of some even-parity charge-transfer exciton components.^b^

**Figure 12:**
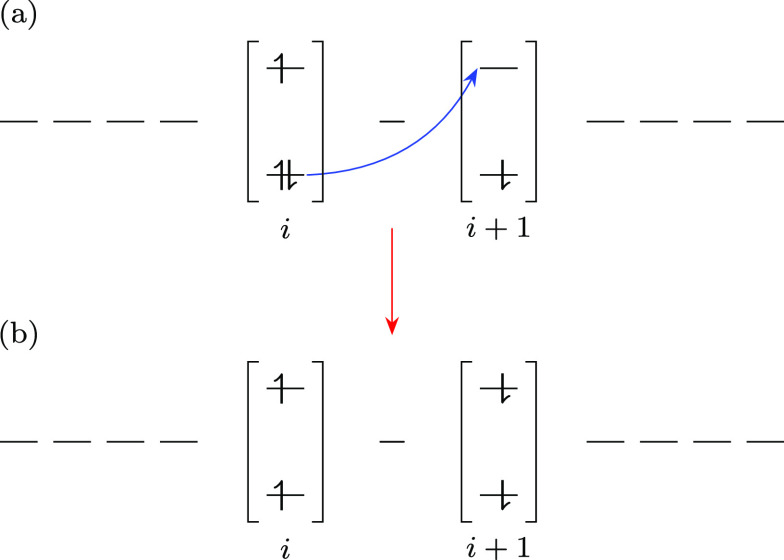
Schematic diagram illustrating the internal conversion of the odd-parity charge-transfer component of the 1^1^B_u_ state (a) to the nearest neighbor singlet triplet-pair component of the 2^1^A_g_ state (b). Note that (a) is not a spin-symmetrized state; see ref (21) for an illustration of correctly spin-symmetrized singlet charge-transfer states.

In practice, because of substituent side groups, carotenoids do not possess definite particle–hole symmetry, and so neither do their electronic states. This means that the 2^1^A_g_ state possesses some charge-transfer exciton components of *even*-parity, while the 1^1^B_u_ state possesses some charge-transfer exciton components of *odd*-parity. As illustrated in Figure 12, it is these odd-parity charge-transfer components of the 1^1^B_u_ state which readily interconvert to singlet triplet-pairs, and thus cause the “bright” to “dark” state internal conversion. In spirit, this is the same mechanism proposed to explain singlet triplet-pair production in acene dimers,^33^ the difference being that acene molecules replace the ethylene dimers of carotenoid chains.

Evidently, the greater the deviation from perfect particle–hole symmetry, the greater the amount of covalent and ionic mixing in the electronic eigenstates. This then implies a larger coupling and a higher internal conversion yield between the bright and dark states. In our model, the deviation from particle–hole symmetry is represented by *Ĥ*_ϵ_ (eq 6) and in particular by the value of {ϵ_*n*_}. In our companion paper, ref (22), we quantify this statement by computing the probabilities of adiabatic and diabatic transitions as a function of {ϵ_*n*_}. As ϵ_*n*_ → 0 the probability of a 1^1^B_u_^+^ to 2^1^A_g_^–^ transition vanishes, while the probability of a S_1_ to S_2_ transition becomes unity. Conversely, for large ϵ_*n*_ the probability of a S_1_ to S_2_ transition vanishes and the probability of a 1^1^B_u_^+^ to 2^1^A_g_^–^ transition increases.

Next, we explain *why* internal conversion happens within 10 fs, i.e., we address the equivalent question of why after photoexcitation there is a diabatic energy-level crossing of the Frenkel exciton and singlet triplet-pair state. The answer to this question lies at the heart of what makes the electronic properties of linear polyenes so fascinating. It involves the roles of both electron–electron interactions and electron–nuclear coupling. We approach this question in three ways.

We first consider the electronic states of linear polyenes in the absence of electron–nuclear coupling, i.e., when electronic interactions dominate. In this case polyenes are Mott–Hubbard insulators: there is a charge-correlation gap between the ground state (i.e., 1^1^A_g_^–^) and the lowest-lying ionic state (i.e., 1^1^B_u_^+^).^12,34^ The ground state is a quantum Heisenberg antiferromagnet with, in the limit of infinitely long chains, a gapless spin density wave or triplet excitation (i.e., the 1^3^B_u_^–^ state). These triplets weakly bind to form gapless singlet triplet-pairs (i.e., the 2^1^A_g_^–^ state). Thus, in this limit there is a large spin-correlation gap between the 1^3^B_u_^–^ and 1^1^B_u_^+^ states, and the “dark” state lies energetically below the “bright” state.

Next, consider electronic states in the absence of electronic interactions, but when electron–nuclear coupling dominates. In this case polyenes are Peierls insulators, i.e., there is a band gap between the filled valence band and the empty conduction band as a result of the incipient bond order wave causing bond dimerization.^12^ Now the 1^3^B_u_^–^ and 1^1^B_u_^+^ states both have an excitation gap and are degenerate. The 2^1^A_g_^–^ state lies higher in energy. As already stated, the ground state is dimerized. The 1^3^B_u_^–^ and 1^1^B_u_^+^ states, however, exhibit solitonic structures and a reversal of the bond dimerization in the middle of the chain from the ground state dimerization. Crucially, the solitons of the triplet (1^3^B_u_^–^ state) are associated with spin-1/2 particles, i.e., spin radicals or spinons, while solitons of the singlet (1^1^B_u_^+^ state) are associated with an electron or hole.^12^

Finally, we consider the intermediate case, relevant for polyenes, when electronic interactions and electron–nuclear coupling are both important. Now, the ground state dimerization is enhanced.^12,35^ The 1^3^B_u_^–^ and 1^1^B_u_^+^ states exhibit a large spin-correlation gap. As a consequence, the 2^1^A_g_^–^ state has significant triplet-pair character, causing the 1^1^B_u_^+^ and 2^1^A_g_^–^ states to be quasidegenerate in the ground state geometry (i.e., at the Franck–Condon point).^21^ The relaxed geometries of the 1^3^B_u_^–^ and 1^1^B_u_^+^ states are now quite different. The relaxed geometry of the 1^3^B_u_^–^ state is similar to the noninteracting limit: there are soliton–antisoliton structures associated with the spin-radicals and a reversal of bond dimerization from the ground state. However, the relaxed 1^1^B_u_^+^ geometry is quite different from the noninteracting limit: as a consequence of the electron–hole interaction, the soliton and antisoliton attract forming an exciton-polaron whose bond dimerization is only slightly different from the ground state. Thus, the 1^3^B_u_^–^ state exhibits a large reorganization energy, while the 1^1^B_u_^+^ state does not. Similarly, the 2^1^A_g_^–^ state, being composed of a triplet-pair, also exhibits a large reorganization energy.

In summary, the reasons that there is an energy level crossing between the diabatic bright and dark states are the following: First, because of the large spin-correlation gap, the dark state has a large triplet-pair component and thus the bright and dark states are quasidegenerate at the Franck–Condon point. Second, the triplet state (1^3^B_u_^–^) has a larger reorganization energy than the 1^1^B_u_^+^ state and consequently, because of its triplet-pair character, the reorganization of the dark state is much larger than for the bright state. Consequently, after photoexcitation to the bright state, nuclear forces cause the level crossing, and hence ultrafast internal conversion to the dark state.

## 4 Exothermic Intermolecular Singlet Fission

As shown in Figure S1 of the Supporting Information, and refs (19 and 20), the intramolecular triplet-pair binding energy varies from ca. 1 eV in short carotenoids to ca. 0.3 eV in long polyene chains. Thus, intramolecular singlet fission is a strongly endothermic process, consistent with the absence of experimental evidence for free triplets on isolated carotenoids generated via a singlet fission mechanism.^36,37^

In this section we address the question of how intermolecular singlet fission can in principle be an exothermic process. Clearly, triplets on single molecules must be energetically stabilized to overcome the intramolecular triplet-pair binding. There are two causes for this. First, there is quantum deconfinement: a single triplet delocalized on a whole molecule has a smaller kinetic (or zero point) energy than a single triplet confined to half a molecule, which is the relevant comparison for two unbound triplets on the same chain. That is, it costs less energy to unbind a pair of triplets on separate molecules than on the same molecule. Second, self-localized triplets on two separate molecules experience a larger (negative) reorganization energy than two bound triplets on the same molecule. Crucially, there are two components to the reorganization energy: a term arising from C–C bond stretches and an additional term arising from torsional relaxation. Therefore, for exothermic intermolecular singlet fission to occur, the molecules must exist in an environment so that they have twisted ground state conformations.

To quantify these statements, we supplement the UVP model (introduced in section 2) by terms that model electron-torsional coupling. The π-electron transfer integral is β(θ) = β_0_ cos θ, where θ is the dihedral angle between neighboring C–H groups. Assuming a small planarization in the excited state, i.e., assuming that δθ ≪ θ^0^, we may write
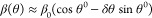
9where θ^0^ is the ground state equilibrium dihedral angle. Thus, −β_0_ sin θ^0^ is the electron-torsional coupling parameter that couples the bond-order operator, *T̂*, to the variation in dihedral angle, δθ. We also assume that there is a harmonic elastic energy
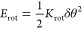
10

Applying the Hellmann–Feynman theorem implies that the equilibrium dihedral angle for the *n*th bond is θ_*n*_ = θ_*n*_^0^ + δθ_*n*_, where
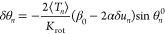
11and δ*u*_*n*_ = (*u*_*n*+1_ – *u*_*n*_) is the change of bond length caused by electronic coupling to C–C bond vibrations.

We define the intermolecular triplet-pair binding energy, Δ*E*_TT_, as twice the energy of triplets on separate molecules relative to the intramolecular triplet-pair state (i.e., 2A_g_), namely,
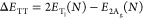
12where *N* is the number of conjugated carbon-atoms in each molecule. A negative value of Δ*E*_TT_ implies exothermic intermolecular singlet fission.

Our results are presented in Tables 1–3. Table 1 shows the results for a pair of carotenoid chains of 22 conjugated carbon-atoms each, for different ground state twists, θ^0^, and torsional force constants, *K*_rot_. Evidently, in the absence of torsional relaxation (e.g., if θ^0^ = 0 or *K*_rot_ → *∞*) intermolecular singlet fission is endothermic. For a fixed *K*_rot_ singlet fission becomes more exothermic for a more twisted molecule in the ground state. Similarly, for a fixed ground state twist, singlet fission becomes more exothermic as *K*_rot_ is reduced.

**Table 1:** Triplet-Pair Binding Energies, Δ*E*_TT_, in meV Defined in eq 12, for a Pair of Carotenoid Chains, Both of 22 Conjugated Carbon Atoms^a^

	*K*_rot_ (eV rad^–2^)			
θ^0^ (deg)	6	8	10	*∞*
0	+102	+102	+102	+102
5	+75	+88	+93	+102
10	–5	+46	+68	+102
15	–134	–22	+27	+102
20		–111	–27	+102

a A negative value implies exothermic intermolecular singlet fission.

Table 2 indicates that for the same values of θ^0^ and *K*_rot_ intermolecular singlet fission becomes less favorable as the number of conjugated carbon atoms increases. This is because the energy gained by quantum deconfinement reduces with increasing chain length.

**Table 2:** Triplet-Pair Binding Energies in meV for a Pair of Carotenoid Chains of *N* Conjugated Carbon Atoms^a^

*N*	binding energy (meV)
18	–54
22	–22
26	+2

a θ^0^ = 15° and *K*_rot_ = 8 eV rad^–2^.

Finally, Table 3 lists the various contributions that favor exothermic intermolecular singlet fission. Quantum deconfinement onto two molecules reduces the intramolecular binding energy on 22-site chains from 781 to 226 meV; bond relaxation causes a 124 meV decrease in binding energy; while additional torsional relaxation causes another 124 meV decrease, rendering the process exothermic. Evidently, quantum deconfinement causes the largest reduction in binding energy, but all three components are necessary to enable exothermic singlet fission. In particular, it is necessary that the molecules are twisted in their ground states for exothermic singlet fission to occur.

**Table 3:** Triplet-Pair Binding Energies in meV for a Pair of Carotenoid Chains of 22 Conjugated Carbon Atoms^a^

intramolecular vertical	intermolecular vertical	bond relaxation	bond and torsional relaxation
781	226	102	–22

a θ^0^ = 15° and *K*_rot_ = 8 eV rad^–2^.

## 5 Discussion and Conclusions

This paper has described our simulations of the excited state dynamics of the carotenoid neurosporene following its photoexcitation into the “bright” (nominally 1^1^B_u_^+^) state. We employed the adaptive tDMRG method on the UV model of π-conjugated electrons and used the Ehrenfest equations of motion to simulate the coupled nuclei dynamics.

To account for the experimental and theoretical uncertainty in the relative energetic ordering of the nominal 1^1^B_u_^+^ and 2^1^A_g_^–^ states at the Franck–Condon point, we considered two sets of parameters. In both cases there is ultrafast internal conversion from the “bright” state to a “dark” singlet triplet-pair state, i.e., to one member of the “2A_g_” family of states.

For one parameter set, internal conversion from the 1^1^B_u_^+^ to 2^1^A_g_^–^ states occurs via the dark intermediate 1^1^B_u_^–^ state. In this case there is a cross over of the 1^1^B_u_^+^ and 1^1^B_u_^–^ diabatic energies within 5 fs and an associated avoided crossing of the S_2_ and S_3_ adiabatic energies. Such an intermediate state has been postulated to explain the violation of the S_2_–S_1_ energy-gap law in carotenoids.^14,15^ Following the adiabatic evolution of the S_2_ state from predominately 1^1^B_u_^+^ character to predominately 1^1^B_u_^–^ character, there is a slower nonadiabatic transition from S_2_ to S_1_, accompanied by an increase in the population of the 2^1^A_g_^–^ state. This scheme is illustrated in Figure 4.

For the other parameter set, the 2^1^A_g_^–^ energy lies higher than the 1^1^B_u_^+^ energy at the Franck–Condon point. In this case there is cross over of the 2^1^A_g_^–^ and 1^1^B_u_^+^ energies and an avoided crossing of the S_1_ and S_2_ energies, as the S_1_ state evolves adiabatically from being of 1^1^B_u_^+^ character to 2^1^A_g_^–^ character. This scheme is illustrated in Figure 8.

We make a direct connection from our predictions to experimental observables by calculating the time-resolved excited state absorption. For the case of direct 1^1^B_u_^+^ to 2^1^A_g_^–^ internal conversion, we showed that the dominant excited-state transition at ca. 2 eV, being close to but lower in energy than the T_1_ to T_1_^*^ transition, can be attributed to the 2^1^A_g_^–^ component of S_1_. Moreover, we show that it is the charge-transfer exciton component of the 2^1^A_g_^–^ state that is responsible for this transition (to a higher-lying exciton state), and not its triplet-pair component. This transition is blue-shifted from the Franck–Condon point, because of the large reorganization energy of the 2^1^A_g_^–^ state.

We next discussed the microscopic mechanism of “bright” to “dark” state internal conversion, emphasizing that this occurs via the exciton components of both states. It is also a fast and efficient process, because the strongly correlated nature of the dark 2^1^A_g_^–^ state means that it has a much larger reorganization energy than the bright 1^1^B_u_^+^ state.

Finally, we described a mechanism whereby the strongly bound intrachain triplet-pairs of the “dark” state may undergo interchain exothermic dissociation. This mechanism relies on the possibility of the unbound interchain triplets being energetically stabilized by quantum deconfinement and larger bond and torsional reorganization energies. We predict that this is only possible if the molecules are twisted in their ground states.

Irrespective of the ordering of the 1^1^B_u_^+^ and 2^1^A_g_^–^ states at photoexcitation, our simulations indicate that after 50 fs the yield of the “dark” predominately singlet triplet-pair states is ca. 65%. This implies, however, that the evolving state still has some “bright” (i.e., 1^1^B_u_^+^) component, which explains the weak emissive character of photoexcited carotenoids.

In an earlier paper we explained the origin of the intrachain triplet-pair binding,^21^ while in this paper we argue that exothermic interchain triplet-pair dissociation is possible if it is accompanied by torsional relaxation. Work is now in progress to build a full model of singlet triplet-pair dissociation and spin decoherence to understand the full kinetic process of singlet fission in carotenoid dimers. Future work will also investigate our model with fully quantized phonons. This will enable us to calculate the vibronic line shape of the photoinduced absorption spectra, in particular allowing for a comparison of the S_1_ and T_1_ transient absorption.^31^ We will also investigate the validity of the Ehrenfest approximation for the nonadiabatic S_2_ to S_1_ transition, discussed in section 3.1.1.

## Supporting Information Available

Parametrization of the UV-Peierls Hamiltonian, parametrization of the symmetry breaking Hamiltonian, *Ĥ*_ϵ_, and probabilities that the adiabatic states, S_1_, S_2_, and S_3_ occupy the diabatic states 2^1^A_g_^–^, 1^1^B_u_^+^, and 1^1^B_u_^–^ for case a (PDF)

## Footnotes

a Readers are referred to ref (19) for a calculation of exciton wave functions in linear polyenes and to ref (12) for a discussion of excitons in conjugated polymers.

b The even (odd)-parity is indicated by the positive (negative) particle–hole symmetry label on the term symbol.
