# Supplementary material for: Photoexcited State Dynamics and Singlet Fission in Carotenoids
Source: J Phys Chem A. 2023 Jan 26;127(5):1342–52. doi: 10.1021/acs.jpca.2c07781 (PMC9923744; doi:10.1021/acs.jpca.2c07781)
Supplement: Supplementary file 1 — jp2c07781_si_001.pdf [file jp2c07781_si_001.pdf]

Photoexcited State Dynamics and Singlet Fission in Carotenoids Supporting Information

Dilhan Manawadu,[*,†,‡] Timothy N. Georges,[†,¶] and William Barford[*,†]

†*Department of Chemistry, Physical and Theoretical Chemistry Laboratory, University of Oxford, Oxford, OX1 3QZ, United Kingdom*

‡*Linacre College, University of Oxford, Oxford, OX1 3JA, United Kingdom*

¶*Brasenose College, University of Oxford, Oxford, OX1 4AJ, United Kingdom*

E-mail: dilhan.manawadu@chem.ox.ac.uk; william.barford@chem.ox.ac.uk

1 Parametrization of the UV-Peierls Hamiltonian

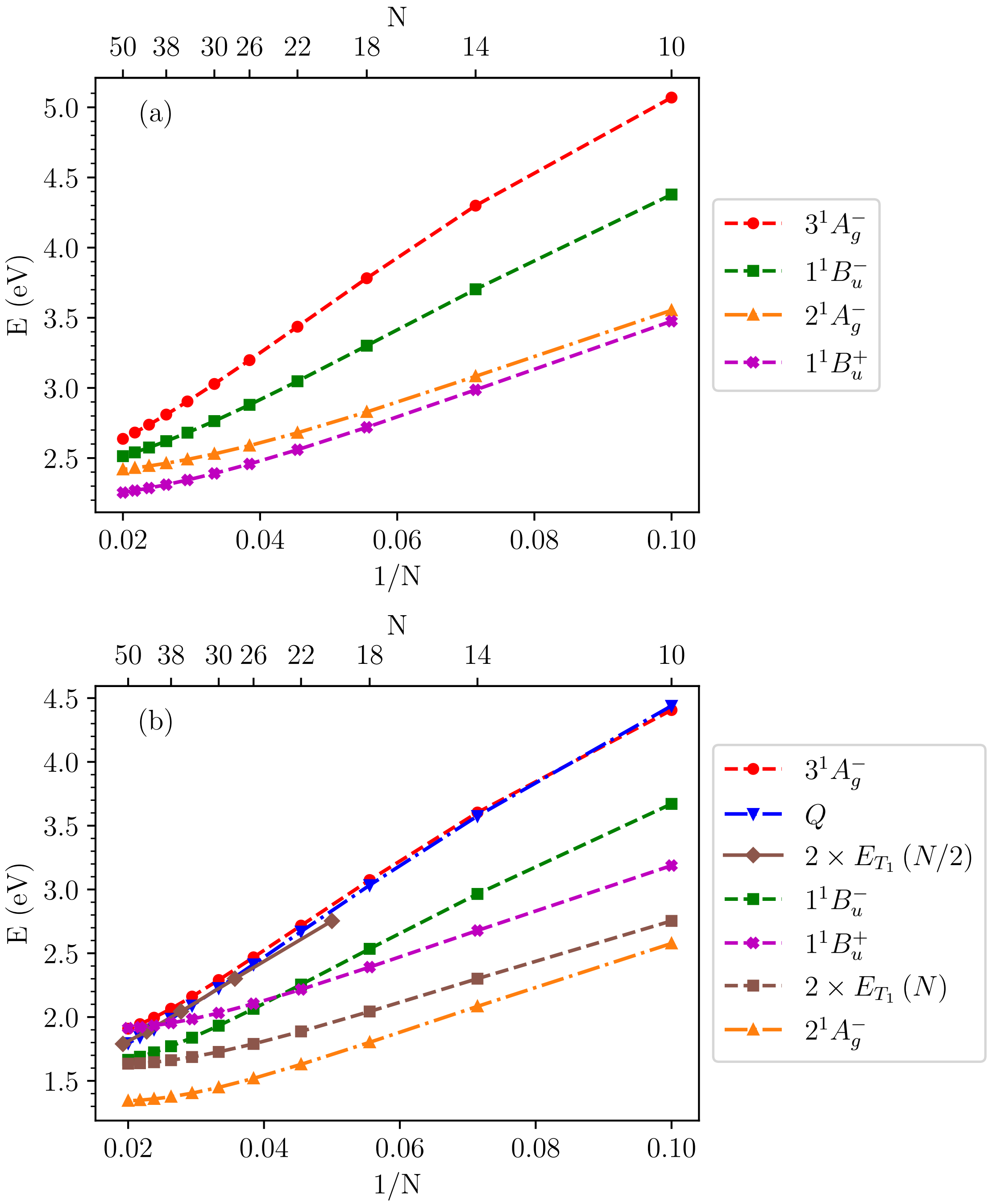

Figure S1: Vertical (a) and relaxed (b) singlet excitation energies of UV-Peierls model with $U = 7.25$ eV and $V = 3.25$ eV, found by solving eq (5) of the main paper. N is the number of conjugated carbon atoms of the system. The vertical energy gaps of ~ 0.1 eV between $1^1B_u^+$ (magenta) and $2^1A_g^-$ (orange) for $18 \leq N \leq 26$ agree with the corresponding excitation energies reported in ref.[1]

Figure S1 illustrates the diabatic vertical and relaxed excitation energies of the UV-Peierls model parametrized for direct internal conversion from the $1^1B_u^+$ state to the $2^1A_g^-$ state. The parametrization is performed such that the vertical excitation energy gap between the $1^1B_u^+$ to the $2^1A_g^-$ states reproduces the excitation energies reported in Table 2 of ref 1.

2 Parametrization of the symmetry breaking Hamiltonian, $\hat{H}_\epsilon$

As described in ref 2, $\hat{H}_\epsilon$ is optimized under the constraint $|\epsilon_n| < \epsilon_{\max}$ such that the ground state π-electron density on C-atom n reproduces the Mulliken charge densities of the π-system found *via* ab initio density functional theory (DFT) calculations. The optimized $\hat{H}_\epsilon$ is given in Table S1. The cut-off $\epsilon_{\max} = 1.0$ is chosen such that $\Psi(t = 0)$ retains sufficient $1^1B_u^+$ character while accurately reproducing the DFT densities with a coefficient of variation $r^2(\boldsymbol{\epsilon}) = 0.92$.

Table S1: The π-electron Mulliken charges from the *ab-initio* DFT calculation, parameters for $\hat{H}_\epsilon$ found for $\epsilon_{\max} = 1.0$ eV, and the expectation values of number densities calculated from the parametrized $\hat{H}_\epsilon$. In order to maintain π-electron charge neutrality, each *ab-initio* charge was increased by $0.05q$. The chemical formula of neurosporene is shown in Figure 1 of the main paper.

		$V = 2.75$ eV		$V = 3.25$ eV	
Carbon site, n	Mulliken charges (q)	ϵ_n (eV)	$\left\langle \hat{N}_n - 1 \right\rangle$	ϵ_n (eV)	$\left\langle \hat{N}_n - 1 \right\rangle$
1	0.14	-1.00	0.17	-1.00	0.17
2	-0.18	0.81	-0.14	0.56	-0.14
3	-0.05	1.00	0.05	1.00	0.06
4	-0.18	1.00	-0.10	0.82	-0.10
5	0.15	-1.00	0.15	-1.00	0.15
6	-0.14	0.27	-0.10	0.02	-0.10
7	-0.07	1.00	0.03	1.00	0.03
8	-0.16	1.00	-0.09	0.84	-0.09
9	0.13	-1.00	0.12	-1.00	0.12
10	-0.09	0.14	-0.07	-0.01	-0.07
11	-0.11	1.00	-0.03	1.00	-0.04
12	-0.10	1.00	-0.02	1.00	-0.03
13	-0.11	0.32	-0.09	0.09	-0.09
14	0.14	-1.00	0.13	-1.00	0.13
15	-0.18	1.00	-0.09	0.92	-0.10
16	-0.05	1.00	0.05	1.00	0.06
17	-0.19	0.90	-0.14	0.61	-0.14
18	0.14	-1.00	0.18	-1.00	0.17

3 Probabilities that the adiabatic states, S_1, S_2 and S_3 occupy the diabatic states $2^1A_g^-$, $1^1B_u^+$ and $1^1B_u^-$

Figure S2 illustrates the probabilities that the adiabatic states S_1, S_2, and S_3 occupy the diabatic states $2^1A_g^-$, $1^1B_u^+$, and $1^1B_u^-$. Adiabatic states are $\sim 90\%$ occupied by the diabatic states at all times. Using the probabilities that the triplet-pair states, $2^1A_g^-$ and $1^1B_u^-$, occupy the adiabatic states, S_1 and S_2, the 'classical' total triplet-pair yield can be calculated via eq (7) of the main paper.

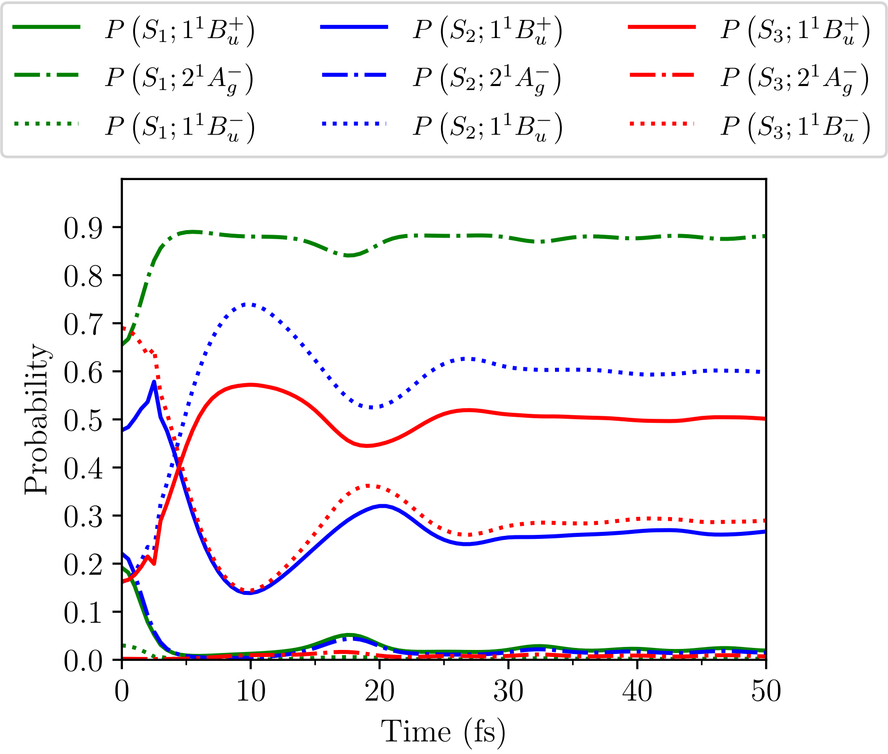

Figure S2: Probabilities as a function of time that the adiabatic states, S_1, S_2 and S_3, occupies the diabatic states, $2^1A_g^-$, $1^1B_u^+$ and $1^1B_u^-$. Results are for neurosporene ($N = 18$) with $V = 2.75$ eV.

References

(1) Taffet, E. J.; Lee, B. G.; Toa, Z. S. D.; Pace, N.; Rumbles, G.; Southall, J.; Cogdell, R. J.; Scholes, G. D. Carotenoid Nuclear Reorganization and Interplay of Bright and Dark Excited States. *The Journal of Physical Chemistry B* **2019**, *123*, 8628–8643.

(2) Manawadu, D.; Valentine, D. J.; Barford, W. Dynamical Simulations of Carotenoid Photoexcited States using Density Matrix Renormalization Group Techniques. 2022; `https://arxiv.org/abs/2211.02022`.
